# PET/CT Is Complementary to Fine-Needle Aspiration Cytology in Assessment of Irradiated Neck in Head and Neck Cancers

**DOI:** 10.1155/2014/191267

**Published:** 2014-05-18

**Authors:** R. C. L. Chan, Y. W. Chan

**Affiliations:** Division of Head and Neck Surgery, Department of Surgery, Queen Mary Hospital, Hong Kong

## Abstract

*Background.* Accurate assessment of irradiated neck in squamous cell carcinoma of the head and neck (HNSCC) is essential. Fine-needle aspiration cytology is often performed for suspicious lesions but it is limited by its low negative predictive value (NPV). We postulated that F-18 fluorodeoxyglucose (FDG) positron emission tomography combined with computed tomography (PET/CT) can overcome this limitation by its high NPV value and allow for a more accurate assessment of irradiated neck in HNSCC. *Methods.* Fifty-four HNSCC patients were included for the study. They all received previous irradiation to the neck. Clinical characteristics, details of radiotherapy, PET/CT results, follow-up findings, and final histological diagnosis were analyzed. *Results.* The sensitivity, specificity, positive predictive value (PPV), and NPV were 95.8%, 96.7%, 95.8%, and 96.7%, respectively. Age, sex, radiation dose, interval between PET/CT and radiotherapy completion, nature of radiotherapy, and use of second course of radiotherapy were not found to affect diagnostic accuracy of PET/CT. A new algorithm for investigation of masses in irradiated neck is proposed. *Conclusions.* PET/CT is an effective diagnostic tool and has a complementary role to FNAC in the management of irradiated neck in head and neck cancers, particularly in cases where suspicious lesions were identified but FNAC showed negative results.

## 1. Background

Radiotherapy is frequently employed in the management of head and neck squamous cell carcinoma (HNSCC). Locoregionally advanced tumours are frequently given adjuvant radiotherapy with or without chemotherapy to improve locoregional control. Definitive radiation is also given for organ preservation and to avoid operative morbidity.

Accurate assessment of irradiated neck is essential in management of HNSCC. Unfortunately, radiation-associated fibrosis of skin and subcutaneous tissue can easily mask underlying lesions on clinical examination. Various modalities of imaging were used to detect residual or recurrent neck diseases. However, anatomical distortion caused by radiotherapy (and often together with surgery) renders that the assessment of disease status by imaging alone is difficult.

Ideally, when a suspicious lesion is identified clinically or radiologically, tissue biopsy should be sampled from suspicious lesions. In reality, however, they are often limited by inaccessibility and/or potential morbidity associated, especially in irradiated necks. Thus, fine-needle aspiration cytology (FNAC) is often performed instead. However, we have recently shown that while its positive predictive value (PPV) is high (89%), negative predictive value (NPV) of FNAC is rather low (37%) in irradiated necks [1]. The low NPV of FNAC necessitated an alternative mean of assessment in these cases.

We know that cancer cells are hypermetabolic with increased glucose metabolism [2]. F-18 fluorodeoxyglucose (FDG), as an analogue of glucose, accumulates in such lesions, allowing their detection by positron emission tomography (PET). A low FDG uptake is suggestive of the absence of cancer tissues, as these tissues, by definition, involve unregulated cell growth which requires a lot of energy from cell metabolism. We can therefore expect a high NPV in PET for detecting malignancy.

On the other hand, various normal structures in the neck region, such as muscles, salivary glands, thyroid glands, and vocal cords, have variable FDG uptake and can lead to false positivity. Benign hypermetabolic processes, such as inflammation, can cause similar confusion in interpretation. The interpretation is even more difficult in patients with previous treatments. For instance, free jejunal flaps used in reconstruction of circumferential pharyngectomy defect can contain hypermetabolic lymphadenopathy in their mesenteric pedicles [3]. Likewise, vocal cord paralysis from previous surgery can cause compensatory effort in the normal cord and result in increased FDG uptake. PPV of PET may be lowered by all these factors.

When performed alone, PET is limited by its spatial resolution and lack of anatomic details which hinder precise tumor localization. Hence, combined PET-computed tomography (PET/CT) is often performed instead to overcome these shortcomings. PET/CT allows for reliable coregistration of anatomical and functional imaging data. It permits accurate tumour localization and facilitates identification of physiologic uptake. As a sole mean of neck assessment, PET/CT may be limited by its PPV and lack of cytopathological diagnosis, but, together with the high PPV of FNAC, these investigations may complement each other and allow for an accurate assessment of irradiated necks.

We reviewed our experience with PET/CT in management of irradiated neck in an attempt to elucidate the diagnostic performance. Other factors that may potentially affect the diagnostic outcomes were also reviewed.

## 2. Methods

A computer search was performed to identify all patients who have been treated under the Division of Head and Neck Surgery, Department of Surgery, Queen Mary Hospital, from January 2008 to December 2011, with histologically proven HNSCC. We retrospectively retrieved all patients who had PET/CT performed. Only patients who received previous irradiation to the neck were included. Nonmalignant cases, cases of cutaneous malignancies, and other types of malignancy (including salivary gland tumours and nasopharyngeal carcinoma) were excluded. Individual case notes were searched manually and electronically to retrieve details on clinical history, radiotherapy, and pathological results.

PET/CT imaging from skull base through the abdomen was performed 60 minutes following an intravenous injection of 7 to 15 mCi FDG and intravenous contrast. These scans were then interpreted by a board-certified radiologist as positive or negative for cervical metastatic disease.

### 2.1. Reference Standard

Whenever histological diagnosis is available (either by excisional biopsy or neck dissection), it was treated as the reference standard. In cases when radiographic evaluation suggested no cervical metastasis and no tissue sampling was performed, negative clinical and radiological follow-up for at least 6 months was considered as evidence of absence of disease.

### 2.2. Exclusion Criteria

Patients were excluded if they met any one of the following criteria: (1) radiotherapy, chemotherapy, or targeted therapy was given during the period between the PET/CT and histological diagnosis; (2) in cases of negative PET/CT finding without tissue sampling, radiotherapy/chemotherapy/targeted therapy was given within 6 months after PET/CT; (3) in cases of negative PET/CT findings without tissue sampling, a follow-up period less than 6 months; (4) no histological diagnosis for cases with positive PET/CT findings.

Patient's age, gender, site of primary tumour, details of radiotherapy, PET/CT results, follow-up findings, and final histological diagnosis were retrieved.

A PET/CT was defined as “positive” when the radiologist suspected metastatic cervical disease based on increased metabolic activity, usually with a SUVmax over 3.

True positive (TP) was defined as positive PET/CT findings with positive final histology. True negative (TN) was defined as negative PET/CT findings with either a negative final histology or a minimum of 6-month negative clinical and radiological follow-up. False negative (FN) referred to cases with negative PET/CT findings but subsequent histological diagnosis of malignancy. False positive (FP) referred to cases with positive PET/CT findings, but subsequent histology showed no malignancy. The sensitivity (TP/[TP + FN]), specificity (TN/[TN + FP]), positive predictive value (TP/[TP + FP]), and negative predictive value (TN/[TN + FN]) were calculated.

Data were analyzed with SPSS version 18.0 (SPSS, Inc., Chicago, IL, USA). Univariate comparisons of diagnostic outcomes were performed with chi-square test for nominal variables and logistic regression for continuous variables. A *P* value of 0.05 or less was considered significant.

## 3. Results

Table 1 summarized the demographics and clinical characteristics of the 54 patients included. The median age at time of PET/CT was 60 years (range 27–88). Male-to-female ratio was 2.4 to 1. The commonest site of primary tumor was the oral cavity, followed by oropharynx and hypopharynx/cervical oesophagus. Six patients had tumours at multiple sites along the head and neck region.

Apart from radiotherapy, 33 patients (66%) had previous neck dissection. Forty-four patients (81%) received previous chemotherapy. The median interval between PET/CT scan and time of completion of radiotherapy was 8 months (range 0–369).

There was only one case of false positive and one case of false negative findings in our series. The overall sensitivity, specificity, PPV, and NPV were 95.8%, 96.7%, 95.8%, and 96.7%, respectively (Table 2).

Age, sex, radiation dose, interval between PET/CT and radiotherapy completion, nature of radiotherapy (primary versus adjuvant treatment), and use of second course of radiotherapy were not found to affect diagnostic accuracy of PET/CT (Table 3).

## 4. Discussion

Assessment of disease status in irradiated necks can be very challenging. Clinical examination often reveals fibrotic neck which can easily mask any underlying lesion. Conventional imaging, such as ultrasound, computed tomography (CT) or magnetic resonance imaging (MRI), is difficult to interpret. Radiotherapy renders indistinct tissue planes and its associated edema in early postradiotherapy period may produce an apparent increase in volume to further complicate interpretation. If there were previous operations as well, neck dissection and various forms of reconstruction can distort normal anatomy and make conventional imaging even more confusing. Rate and lesion of lesion growth have been used by many as the criteria for recurrence, but it has been shown that such growth can occur as normal postirradiation effect [4]. On the other hand, in actual cases of recurrence, such approach potentially delays diagnosis and treatment.

PET/CT with FDG has been shown to be useful in diagnosing, staging, and restaging HNSCC [5]. Reduced FDG uptake was shown to correlate with a reduction in the number of viable tumor cells in early study [6]. PET as a mean for staging HNSCC has shown promising results with sensitivity and specificity for detecting cervical lymph node involvement up to 90% and 96%, respectively [7, 8]. The greatest strength of PET/CT is its ability to evaluate metabolic changes, independent of the lesion size. Such properties are particularly useful in detecting residual or recurrent disease after treatment. PET was reported to have sensitivities and specificities in the ranges of 96% –100% and 72% – 93% [9, 10], respectively, for surveillance of recurrent HNSCC.

Our study has shown that PET/CT has high PPV (95.8%) and NPV (96.7%) in irradiated neck irrespective of nature of treatment (primary RT versus adjuvant RT), dosage, or interval between PET/CT and RT completion. Given its superior diagnostic ability over most conventional imaging modalities, PET/CT should ideally be employed in all HNSCC patients requiring assessment of irradiated neck. In reality, however, the need for neck assessment after irradiation is not uncommon in HNSCC patients and funding for a universal PET/CT assessment policy is impractical due to its high cost, at least in our locality and in most developing countries. There is limited evidence in current literature on economic evaluation of PET/CT in HNSCC patients. Studies have been conducted to evaluate roles of PET/CT in detecting occult neck metastasis in N0 patients [11], detecting distant metastasis in advanced disease [12, 13], and restaging recurrent laryngeal cancers [14]. To the best of our knowledge, no study has been performed to evaluate the cost-effectiveness of PET/CT in assessment of irradiated neck in HNSCC patients.

To take advantage of the diagnostic accuracy of PET/CT in limited resources, a realistic diagnostic strategy is necessary to selectively offer PET/CT to those most in need of it and substitute PET/CT with more affordable alternatives in the remaining patients. We have recently shown that FNAC for irradiated neck masses is a safe, cheap, and effective diagnostic tool with a PPV up to 89%. However, it was limited by its unacceptably low NPV (37%) [1]. In our locality, a PET/CT would cost around $3,000, whereas a cytological evaluation of FNAC specimen would cost only around $100. FNAC, with ultrasound guidance if necessary, is performed by clinicians, most of the time in our centre.

**Figure 1:**
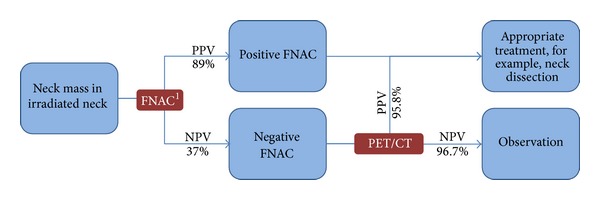
Proposed algorithm for investigation of neck mass in irradiated neck. See [1].

In an attempt to strike a balance between cost and diagnostic accuracy, we proposed a new algorithm for investigation of masses in irradiated neck taking advantages of both tests (Figure 1). All masses in irradiated necks are subjected to FNAC assessment initially. When FNAC shows a positive finding, treatment should be given accordingly as FNAC has a high PPV. However, when FNAC shows a negative result, malignancy cannot be reliably ruled out since the NPV of FNAC is low (37%). PET/CT is then performed in these patients. Should the PET/CT be negative, its high NPV (96.7%) will give clinicians more confidence in taking a conservative approach. On the other hand, if the PET/CT turns out to be positive, its high PPV (95.8%) may justify a more aggressive mean in obtaining a pathologic diagnosis, such as an excisional biopsy. With this algorithm, we believe that PET/CT and FNAC complement each other in the management of irradiated neck in head and neck cancers.

Our study is limited by its retrospective nature. Pathological diagnosis was not available in all patients. Occasionally, patients with positive radiological findings with gross clinical sign of advanced malignancy were decided for palliative care. Tissue biopsies are not taken from these patients to avoid unnecessary suffering. These patients represent a group of “true positive” data that we have excluded. Excluding them may have lowered the PPV.

## 5. Conclusion

PET/CT is an effective diagnostic tool and has a complementary role to FNAC in the management of irradiated neck in head and neck cancers. In particular, it is useful in guiding management in cases where suspicious lesions were identified, but FNAC showed negative results.

## Figures and Tables

**Table 1:** Patient demographics and clinical characteristics (*N* = 54).

Median age (range)	60 (27–88)
Sex (M : F)	2.4 : 1
Site of primary tumour	
Oral cavity	30 (55%)
Oropharynx	9 (17%)
Hypopharynx/cervical oesophagus	9 (17%)
Multiple sites	6 (11%)
Previous treatment	
Neck dissection	33 (61%)
Chemotherapy	44 (81%)

**Table 2:** Overall accuracy of PET/CT in irradiated neck.

Positive predictive value (PPV)	95.8%
Negative predictive value (NPV)	96.7%
Sensitivity (SN)	95.8%
Specificity (SP)	96.7%

**Table 3:** Factors affecting diagnostic accuracy of PET/CT in irradiated neck.

	Sensitivity	Specificity	PPV	NPV
Sex	.699	.374	.699	.374
Age	.652	.354	.507	.491
RT dose	.906	.958	.622	.191
Interval between PET/CT and RT completion (months)	.578	.759	.099	.626
Nature of RT (primary versus adjuvant treatment)	.328	.460	.328	.193
Second course of RT	.470	.850	.470	.850

RT: radiotherapy; PPV/NPV: positive/negative predictive value.
